# Building Process-Oriented Data Science Solutions for Real-World Healthcare

**DOI:** 10.3390/ijerph19148427

**Published:** 2022-07-10

**Authors:** Carlos Fernandez-Llatas, Niels Martin, Owen Johnson, Marcos Sepulveda, Emmanuel Helm, Jorge Munoz-Gama

**Affiliations:** 1 SABIEN—Institute of Information and Communication Technologies (ITACA), Universitat Politecnica de Valencia, Camino de Vera S/N, 46022 Valencia, Spain; 2 Department of Clinical Science, Intervention and Technology (CLINTEC), Karolinska Institutet, 171 77 Stockholm, Sweden; 3 Research Group Business Informatics, Hasselt University, Martelarenlaan 42, 3500 Hasselt, Belgium; 4 Research Foundation Flanders (FWO), Egmontstraat 5, 1000 Brussels, Belgium; 5 School of Computing, Faculty of Engineering, University of Leeds, Leeds LS2 9JT, UK; o.a.johnson@leeds.ac.uk; 6 Department of Computer Science, School of Engineering, Pontificia Universidad Católica de Chile, Av. Vicuña Mackenna 4860, Santiago 7820436, Chile; marcos@ing.puc.cl (M.S.); jmun@uc.cl (J.M.-G.); 7 School of Informatics, Communications and Media, University of Applied Sciences Upper Austria, Softwarepark 11, 4332 Hagenberg, Austria; emmanuel.helm@fh-hagenberg.at

**Keywords:** process-oriented data science, process mining, healthcare, artificial intelligence, COVID-19

## Abstract

The COVID-19 pandemic has highlighted some of the opportunities, problems and barriers facing the application of Artificial Intelligence to the medical domain. It is becoming increasingly important to determine how Artificial Intelligence will help healthcare providers understand and improve the daily practice of medicine. As a part of the Artificial Intelligence research field, the Process-Oriented Data Science community has been active in the analysis of this situation and in identifying current challenges and available solutions. We have identified a need to integrate the best efforts made by the community to ensure that promised improvements to care processes can be achieved in real healthcare. In this paper, we argue that it is necessary to provide appropriate tools to support medical experts and that frequent, interactive communication between medical experts and data miners is needed to co-create solutions. Process-Oriented Data Science, and specifically concrete techniques such as Process Mining, can offer an easy to manage set of tools for developing understandable and explainable Artificial Intelligence solutions. Process Mining offers tools, methods and a data driven approach that can involve medical experts in the process of co-discovering real-world evidence in an interactive way. It is time for Process-Oriented Data scientists to collaborate more closely with healthcare professionals to provide and build useful, understandable solutions that answer practical questions in daily practice. With a shared vision, we should be better prepared to meet the complex challenges that will shape the future of healthcare.

## 1. COVID-19 as an Eye-Opener

The COVID-19 pandemic has shaken the world [1]. In the healthcare domain, the pandemic has led to fundamental changes in the way that healthcare is delivered to patients [2], it has influenced the education of future doctors [3], and has highlighted the importance of safeguarding the mental health of professionals when healthcare systems are over-stressed [4], in addition to many other issues. Despite the seriousness of the situation and the volume of resources invested in dealing with the pandemic, there remain unsolved difficulties determining appropriate diagnostics, prognostics and better treatment plans, as well as uncertainty over the best policies for preventing the harm caused by the disease [5]. This situation has demonstrated that it is crucial to rethink the way that we use scientific evidence to solve real and urgent problems, including the scarcity of resources, the economic and societal impact of mismanagement during the health crisis, and the need for a better framework in research ethics in health data research [6,7].

The COVID-19 pandemic has worsened the situation, as overworked medical staff struggled to follow protocols that frequently changed in response to unfolding events [2]. The introduction of new prevention measures prior to medical contact, the decrease of face to face meetings in favor of consultations by telephone, the increase of psychological issues and fears among medical staff, and the changing way in which doctors addressed the diagnosis and treatments of patients are examples of how profoundly medical processes changed. COVID-19 fundamentally influenced the processes by which healthcare was delivered [2]. The prevention measures that were applied affected all the hospital services protocols. For example, the increase in waiting lists in many non-urgent surgeries [8,9] is expected to have a serious impact on the health of millions of patients worldwide.

Science has tried to provide responses to pressing problems caused by the pandemic as quickly as possible. The number of scientific publications about COVID-19 over the last two years has been impressive [10]. This is not only due to the large number of researchers who have re-oriented their research to try to support the fight against the pandemic, but also because publishers and reviewers have increased their efforts to shorten publication times in order to get evidence published as fast as possible [10]. As a result, there is a growing body of literature attempting to summarize the lessons learned during the pandemic [6,7,11].

The extensive literature in the field of Information and Communication Technologies (ICT) applied to healthcare supports a general consensus that ICT has the potential to significantly improve healthcare delivery [12]. As ICT has become more widely adopted, the quantity of data generated by ICT applications has grown exponentially. During the pandemic, data-driven technologies were used to develop a clear view of the effects of COVID-19 and learn the lessons from different national responses [11]. However, even national governments struggled to assess the real impact of COVID-19, including the apparently simple task of computing mortality [13]. Established ICT systems were often inadequate and unable to quickly provide good quality data [14]. On one hand, laws and privacy restrictions prevented researchers from using all the available data and, on the other hand, data was gathered under stressful conditions, with frequent revisions of protocols inevitably affecting data quality.

COVID-19 could be a game-changer with respect to the creation of awareness that data is crucial to healthcare operations. It was a clear demonstration of how valuable data is in order to optimize protocols that ensure the best quality of care beyond the economical and societal frontiers. However, most problems regarding data gathering during the pandemic were not new, the pandemic simply exposed the inadequacy of existing systems. One of the key problems is the poor adoption and use of Electronic Health Record (EHR) systems that are genuinely interoperability and available to support all stages of the patient care [15]. However, this is not the only critical problem. Data is gathered by doctors, nurses and other healthcare professionals who have reported that the use of EHRs increases their workload. It is argued that the use of EHRs can decrease the time available to spend with patients as professionals are pushed to record data on the system [16]. This pressure has an impact in data. In this scenario, the low acceptance of the EHR directly affects the quality of data [17] and, consequently, all the intelligence and the evidence that are potentially achievable from it.

If we want to take advantage of the Artificial Intelligence paradigm in the field of medicine, it will be crucial to incorporate it into the medical workflow in a way that doctors do not consider an additional burden. This requires providing added value to the hospital information systems that doctors consider valuable for their daily practice. The benefits of the EHRs should go beyond a simple evolution from a paper-based history to the digitalization of documents. Real Digital Health transformation should have a deep change on the organizational aspects of hospitals and healthcare [18]. This should include taking advantage of the benefits of having high-quality EHR data for research and enabling a new era for supporting health professionals by collecting evidence from data.

## 2. The Process Oriented Data Science Solution for Healthcare

The Artificial Intelligence revolution in medicine is coming. There exists global consensus that the use of Artificial intelligence will play an important role in the future of medicine [19]. During the pandemic, lots of work appeared based on applying Artificial Intelligence and data-driven techniques to analyze data and support health professionals and citizens in the fight against COVID-19. However, although this demonstrated the possibilities of Artificial Intelligence in healthcare, the real impact in COVID-19 was insufficient. The Artificial Intelligence systems have had serious limitations in the COVID-19 fight [20,21].

We should be aware that we are dealing with Real World Data (RWD) [22]. Real World Data is data collected from routine, daily practice rather than research-focused Randomized Controlled Clinical Trials (RCTs). This data eminently originates from heterogeneous data sources; some is structured, some is not; some is of good quality and complete, some is not; etc. There are many challenging quality problems associated with RWD that should be addressed urgently, and these will certainly affect the future of Artificial Intelligence in medicine [14].

Some of the most evident problems in the digital transformation of healthcare through Artificial Intelligence are poor quality data, poor acceptance of Artificial Intelligence methods by medical professionals, and uncertainty over how these techniques should be applied in the healthcare domain. Against this background, clinical methodologies like Value Based Healthcare [23] or Lean Healthcare [24] focus on the continuous improvement of healthcare processes. The aim of these methods is to improve the process of care by incorporating new ways of thinking within the daily workflow of health professionals.

The Process Oriented Data Science community has actively been developing techniques to support the data-driven improvement of healthcare processes. In recent years, the Process Mining community has been very active in the analysis of healthcare workflows and the identification of current challenges [25,26,27,28]. We have identified a need to bring together the best efforts made by the community to ensure that promised improvements to care processes can be achieved in real healthcare.

From the Artificial Intelligence perspective, it is necessary to provide appropriate tools to support medical experts. To ensure that these tools are fit-for-purpose, interactive communication between medical experts and data scientists is needed to co-create solutions [29]. The active involvement of medical doctors is crucial.

Many of the problems regarding data quality are due to the lack of involvement of experts. In our vision, there is a need to provide instant feedback in daily practice to re-enforce the experienced value of correct data to the health professionals who create it. If they don’t experience the utility of good quality data within their own work, they will not be motivated to improve that quality. For that, it is not only needed to provide good Decision Support Systems, but also, it is necessary to provide tools for supporting health professionals in the correct classification and annotation of data, allowing them to detect the data errors in daily practice [30]. Process Mining techniques can offer solutions for the analysis of patient pathways, providing a useful visualization of current patterns of care. The effective use of Process Mining tools can demonstrate that technology is not a burden for health professionals, but a way to know more about their patients, at all stages of the process.

The use of Artificial Intelligence in daily practice will help health professionals extract evidence from data. However, to do this safely requires understandability of Artificial Intelligence outcomes. That means not only the explainability of the decisions taken by machines, but also helping experts gain an enhanced view of the care process so that they can co-create alternative treatments and diagnosis methods, use tools to predict outcomes, and evaluate the effects of new pathways. Process-Oriented Data-driven solutions should be focused on the process by offering the information necessary for understanding, implementing, and adapting the process and continuously improve clinical protocols.

In our vision, Process-Oriented Data Science, and specifically concrete techniques as Process Mining can offer an easy to manage tools that provide understandable and explainable Artificial Intelligence solutions. Process Mining can offer data-driven solutions that involve the expert in the process of the co-discovery of evidence in an interactive way. The creation of tools that can cover the requirements of doctors’ daily practice is crucial to create useful methods that will be fully accepted. Now is the time for Process-Oriented Data Scientists to collaborate with health professionals to build useful, understandable solutions that answer real questions in daily practice. With a shared vision, we should be better prepared to jointly tackle the complex challenges that will shape the future of healthcare.

## References

[1] Khanna R.C., Cicinelli M.V., Gilbert S.S., Honavar S.G., Murthy G.V.S. (2020). COVID-19 pandemic: Lessons learned and future directions. Indian J. Ophthalmol..

[2] Chang W.H. (2020). The influences of the COVID-19 pandemic on medical service behaviors. Taiwan. J. Obstet. Gynecol..

[3] Rose S. (2020). Medical Student Education in the Time of COVID-19. JAMA.

[4] Park S.S. (2021). Caregivers’ mental health and somatic symptoms during COVID-19. J. Gerontol. Ser. B.

[5] Fang F.C., Benson C.A., del Rio C., Edwards K.M., Fowler V.G., Fredricks D.N., Limaye A.P., Murray B.E., Naggie S., Pappas P.G. (2021). COVID-19—Lessons Learned and Questions Remaining. Clin. Infect. Dis..

[6] Khoo E.J., Lantos J.D. (2020). Lessons learned from the COVID-19 pandemic. Acta Paediatr..

[7] Ruiu M.L. (2020). Mismanagement of Covid-19: Lessons learned from Italy. J. Risk Res..

[8] Domínguez-Gil B., Coll E., Fernández-Ruiz M., Corral E., del Río F., Zaragoza R., Rubio J.J., Hernández D. (2020). COVID-19 in Spain: Transplantation in the midst of the pandemic. Am. J. Transplant..

[9] Garcia-Rojo E., Manfredi C., Santos-Perez-de-la Blanca R., Tejido-Senchez A., Garcia-Gomez B., Aliaga-Benítez M., Romero-Otero J., Rodriguez-Antolin A. (2021). Impact of COVID-19 outbreak on urology surgical waiting lists and waiting lists prioritization strategies in the post-COVID-19 era. Actas Urol. Esp. (Engl. Ed.).

[10] Horbach S.P.J.M. (2020). Pandemic publishing: Medical journals strongly speed up their publication process for COVID-19. Quant. Sci. Stud..

[11] Kuhl E. (2020). Data-driven modeling of COVID-19—Lessons learned. Extrem. Mech. Lett..

[12] Mamlin B.W., Tierney W.M. (2016). The Promise of Information and Communication Technology in Healthcare: Extracting Value from the Chaos. Am. J. Med. Sci..

[13] Lloyd-Sherlock P., Sempe L., McKee M., Guntupalli A. (2021). Problems of Data Availability and Quality for COVID-19 and Older People in Low- and Middle-Income Countries. Gerontologist.

[14] Saez C., Romero N., Conejero J.A., Garcia-Gomez J.M. (2021). Potential limitations in COVID-19 machine learning due to data source variability: A case study in the nCov2019 dataset. J. Am. Med. Inform. Assoc..

[15] Pryor R., Atkinson C., Cooper K., Doll M., Godbout E., Stevens M.P., Bearman G. (2020). The electronic medical record and COVID-19: Is it up to the challenge?. Am. J. Infect. Control.

[16] Rathert C., Porter T.H., Mittler J.N., Fleig-Palmer M. (2019). Seven years after Meaningful Use: Physicians’ and nurses’ experiences with electronic health records. Health Care Manag. Rev..

[17] Joukes E., Keizer N.F.D., Bruijne M.C.D., Abu-Hanna A., Cornet R. (2019). Impact of Electronic versus Paper-Based Recording before EHR Implementation on Health Care Professionals’ Perceptions of EHR Use, Data Quality, and Data Reuse. Appl. Clin. Inform..

[18] Kraus S., Schiavone F., Pluzhnikova A., Invernizzi A.C. (2021). Digital transformation in healthcare: Analyzing the current state-of-research. J. Bus. Res..

[19] Ahuja A.S. (2019). The impact of artificial intelligence in medicine on the future role of the physician. PeerJ.

[20] Chen J., See K.C. (2020). Artificial Intelligence for COVID-19: Rapid Review. J. Med. Internet Res..

[21] Naudé W. (2020). Artificial Intelligence against Covid-19: An Early Review.

[22] Miani C., Robin E., Horvath V., Manville C., Cave J., Chataway J. (2014). Health and Healthcare: Assessing the Real World Data Policy Landscape in Europe.

[23] Gray M. (2017). Value based healthcare. BMJ.

[24] Costa L.B.M., Godinho Filho M. (2016). Lean healthcare: Review, classification and analysis of literature. Prod. Plan. Control.

[25] Munoz-Gama J., Martin N., Fernandez-Llatas C., Johnson O.A., Sepúlveda M., Helm E., Galvez-Yanjari V., Rojas E., Martinez-Millana A., Aloini D. (2022). Process mining for healthcare: Characteristics and challenges. J. Biomed. Inform..

[26] Martin N., De Weerdt J., Fernández-Llatas C., Gal A., Gatta R., Ibáñez G., Johnson O., Mannhardt F., Marco-Ruiz L., Mertens S. (2020). Recommendations for enhancing the usability and understandability of process mining in healthcare. Artif. Intell. Med..

[27] Gatta R., Vallati M., Fernandez-Llatas C., Martinez-Millana A., Orini S., Sacchi L., Lenkowicz J., Marcos M., Munoz-Gama J., Cuendet M. (2019). Clinical Guidelines: A Crossroad of Many Research Areas. Challenges and Opportunities in Process Mining for Healthcare. Proceedings of the Business Process Management Workshops.

[28] De Roock E., Martin N. (2022). Process mining in healthcare–An updated perspective on the state of the art. J. Biomed. Inform..

[29] Fernandez-Llatas C. (2021). Interactive Process Mining in Healthcare.

[30] Martin N., Martinez-Millana A., Valdivieso B., Fernández-Llatas C., Di Francescomarino C., Dijkman R., Zdun U. (2019). Interactive Data Cleaning for Process Mining: A Case Study of an Outpatient Clinic’s Appointment System. Proceedings of the Business Process Management Workshops.

